# Spontaneous Coronary Artery Dissection in Clinical Practice: Pathophysiology and Therapeutic Approaches

**DOI:** 10.3390/medicina60020217

**Published:** 2024-01-26

**Authors:** Andrea D’Amato, Marco Valerio Mariani, Silvia Prosperi, Lorenzo Colombo, Andrea De Prisco, Carlo Lavalle, Massimo Mancone, Carmine Dario Vizza, Paolo Severino

**Affiliations:** Department of Clinical, Internal, Anesthesiology and Cardiovascular Sciences, Sapienza University of Rome, Viale del Policlinico, 00161 Rome, Italy; damatoandrea92@gmail.com (A.D.); silvia.prosperi@uniroma1.it (S.P.); colombolorenzo90@yahoo.it (L.C.); deprisco.1843735@studenti.uniroma1.it (A.D.P.); carlolavalle@yahoo.it (C.L.); massimo.mancone@uniroma1.it (M.M.); dario.vizza@uniroma1.it (C.D.V.); paolo.severino@uniroma1.it (P.S.)

**Keywords:** spontaneous coronary artery dissection, MINOCA, pathophysiology, therapy, percutaneous coronary intervention, coronary artery bypass graft

## Abstract

Spontaneous coronary artery dissection (SCAD) is a cause of myocardial infarction without obstructive coronary artery disease (MINOCA). It is determined by a coronary artery wall layers separation, which occurs regardless of traumatic or iatrogenic injuries. Even if it is often a missed diagnosis, its incidence is growing along with the improvement of intracoronary imaging techniques that allow for better detection. The main angiographical classification distinguishes three different forms, with slightly different prognoses at long-term follow up. SCAD is a recurrent condition, severely hampering the life quality of affected patients. The predominantly young age of patients with SCAD and the high prevalence of females among them have made the topic increasingly important, especially regarding therapeutic strategies. According to the data, the most recommended treatment is conservative, based on the use of antiplatelet agents and supportive anti-ischemic therapy. However, there are conflicting opinions concerning the need for dual antiplatelet therapy and its duration. In the case of invasive treatment, the choice between percutaneous coronary intervention and coronary artery bypass graft depends on the patient’s clinical stability and the interested vessel. The purpose of the current review is to revise the pathophysiological mechanisms underlying SCAD and the current knowledge of its treatment.

## 1. Introduction

Myocardial infarction with non-obstructive coronary arteries (MINOCA) defines the clinical situation of a patient presenting symptoms of an acute coronary syndrome (ACS) with a cardiac troponin elevation in the absence of a significant obstruction of the coronary arteries in invasive angiography [1]. The presence of cardiac symptoms with the elevation of a cardiac biomarker is diagnostic of an acute myocardial injury (AMI), considering the contemporary fourth universal definition of myocardial infarction [2]. After the exclusion of coronary plaques ≥50% in any major epicardial vessel via angiography and an alternative diagnosis has been ruled out (especially pulmonary embolism and myocarditis), MINOCA is identified [2,3].

Almost 10% of patients with ST elevation myocardial infarction (STEMI) do not have evident coronary plaques in invasive angiography [4]. The prevalence of MINOCA in patients with ACS undergoing invasive angiography ranges from 1% to 14% [5]. In particular, the COAPT study showed MINOCA in 5.8% of patients with AMI, while the GENESIS-PRAXY trial identified MINOCA in 8.2% of its sample [6,7]. Nowadays, MINOCA demonstrates a complex pathophysiology encompassing both atherosclerotic and non-atherosclerotic mechanisms. Atherosclerotic mechanisms are clearly related to plaque disruption (i.e., plaque rupture, plaque erosion, and calcific nodules), which can lead to atherothrombosis and eventually AMI. Approximately one-third of MINOCA patients demonstrate plaque disruption in intravascular ultrasound (IVUS) during invasive angiography. Non-atherosclerotic mechanisms mainly include epicardial coronary vasospasm, coronary microvascular dysfunction, coronary embolism/thrombosis, spontaneous coronary artery dissection (SCAD), and supply/demand mismatch. SCAD causes MINOCA due to vessel luminal obstruction, even in cases where the obstruction may not be evident, especially when occurring at distal epicardial vessels [3].

SCAD consists of a coronary artery wall layers separation, which occurs independently of traumatic or iatrogenic injuries. It is a rare and underdiagnosed disease that mainly affects young women without cardiovascular risk factors. Several registries reported an incidence rate ranging from 23% to 36% of myocardial infarction presentation in patients aged under 60 years old [8,9,10,11,12]. However, its prevalence is also increasing in older women [13]. SCAD’s clinical presentation may be varied. It represents 1.7–4% of acute coronary syndrome (ACS) cases. In particular, patients affected by SCAD present mainly with STEMI in 33–87% of cases, and NSTEMI in 13–67% of cases, according to the registry considered [13,14,15,16]. Lobo et al. [17] reported an increased prevalence of left anterior dominant (LAD) and left main involvement with a consequent cardiogenic shock in patients with STEMI related to SCAD compared to patients with atherosclerosis-related STEMI. SCAD represents a frequent cause of MINOCA [17,18]. For this reason, it should always be suspected in women aged under 50 years old meeting the diagnostic criteria for myocardial infarction [2,18]. In 0.5% of cases, SCAD’s clinical onset is sudden cardiac death [19], while ventricular tachycardia and fibrillation have been observed in 3.6% and 11.8% of patients. Cardiogenic shock has been observed in 1.2–15.9% of patients [14,15,16,17,18,19]. The main presentation symptom is chest pain, which is more frequent than in atherosclerosis-related myocardial infarction [20]. This may be due to the presence of dissection, which is an autonomous cause of pain, beyond ischemia. Chest pain is frequently irradiated to the neck and left arm and associated with gastrointestinal-related symptoms. Also, atypical chest pain has been described, particularly a retrosternal burning sensation [21].

The aim of the current review is to comprehensively revise the pathophysiology and current management approach to SCAD.

## 2. Pathophysiology of Spontaneous Coronary Artery Dissection

The pathophysiology of SCAD and its management is different to type 1 AMI. The latter is based on the well-known atherosclerotic plaque disruption complicated by coronary atherothrombosis [2,22]. Regarding SCAD, instead, two main pathogenetic mechanisms have been described: (i) a mechanism concerning the more external arterial wall layers characterized by a possible vasa vasorum rupture with consequent arterial wall bleeding; (ii) a mechanism starting from the inner wall layers characterized by intima tearing and consequent false lumen formation with media hemorrhage and true lumen compression. Margaritis et al. [23] studied histopathological alterations occurring in SCAD-surviving patients versus patients who died. They did not find myocardial necrosis in autopsies, and the inflammatory response is a response to injury and not a primitive mechanism leading to SCAD [23]. Although vasa vasorum may be involved in SCAD pathogenesis, their density is not increased. There are several predisposing factors to SCAD despite the fact that in most cases, its etiopathogenetic mechanism remains an issue. Female gender is the most well-known and strongest predisposing factor, accounting for up to 90% of SCAD cases. In particular, pregnancy, multiparity, peri and post-partum status, and hormonal therapy are major risk factors for SCAD, suggesting a key role for sex-related hormones. Moreover, SCAD occurring during pregnancy appears more severe, involving main and proximal arteries, compromising the left ventricular function and requiring invasive treatment [24]. Up to 72% of patients with SCAD reported fibromuscular dysplasia [13,25], particularly its multifocal clinical presentation [8]. In this regard, the thought that SCAD may represent a complication of fibromuscular dysplasia is reasonable [25]. However, Margaritis et al. [23] reported no histological signs of coronary fibromuscular dysplasia. Also, inherited connective disorders are associated with SCAD, albeit in a minority of cases. Other authors found a close relationship between SCAD and psychoemotional stress [26,27], while intense physical training, such as bodybuilding, may represent a precipitating cause of SCAD, particularly in men [28]. Fahmy et al. [28] reported differences among men and women regarding the role of emotional stress. In fact, emotional stress seems to have a greater weight for women than man [28]. Also, systemic inflammatory diseases, such as systemic lupus erythematosus, have been associated with SCAD [15,29]. However, results have been contrasting, suggesting a non-inflammatory pathogenesis for SCAD [30] and the necessity of further studies on this topic. In conclusion, genetic predisposition to SCAD represents an interesting field of study, for which there are not clear evidence.

## 3. The Role of Genetic Susceptibility in the Spontaneous Coronary Artery Dissection Pathogenesis

The genetic substrate in SCAD pathogenesis is complex, polygenic, and not yet completely understood. Both rare and common disease-associated variants have been found in several genes, which are commonly caused by single nucleotide polymorphisms (SNPs), insertions or deletions, structural variants, intronic variants, and/or short tandem repeat expansions [31,32,33,34,35,36]. Most patients show multigenic rather than monogenic heritage contributing to the pathogenesis of both sporadic and familial SCAD [31,32,33,34]. According to the data, the frequency of genetic variants detected in SCAD patients is almost 8–10%, but it may be underreported [31,36]. Genetic modifiers and environmental modifiers are responsible for variable expression and incomplete penetrance in familiar SCAD [32,37].

An overlap between SCAD-related genes and connective tissue disorder (CTD)-related genes or vasculopathy-related genes has been detected, even in the absence of any major clinical aspect. Most common CTD are linked to genes encoding for extracellular matrix proteins in syndromic patterns, like vascular Ehlers–Danlos syndrome with collagen type III alpha 1 chain (COL3A1) gene, Marfan syndrome with fibrillin-1 (FBN1) gene, or Loeys–Dietz syndrome with transforming growth factor beta receptor I and II (TGFBR1 and TGFBR2), or mothers against decapentaplegic homolog 3 (SMAD3) genes. SCAD can be seen as a component of a complex spectrum in these disorders caused by arterial fragility, even in subclinical cases [31,32,33,36,37,38]. Henkin et al. [39] estimated a 5.1% overlap of SCAD patients with CTD; common physical findings were non-specific joint hypermobility, translucent skin, myopia, and arachnodactyly. Tarr et al. [31] identified the highest presence of CTD-related genes in SCAD patients—almost 11%, when commonly, 3.6–8.2% is reported.

SCAD-related genes are linked to the architecture of the cytoskeleton. In particular, cell–cell adhesion proteins, cell–extracellular matrix adhesion protein (i.e., NOTCH1, COL3A1, COL4A1, COL4A2, and COL5A2), and extracellular matrix remodeling through interactions with the estrogenic receptor have been involved. Also, nuclear factor-κB (NF-κB), metalloproteinases, retinoic acid receptor, and TGF-β signalling pathways, whose disruption is frequently associated with CTD and vasculopathies, have been involved [31,35,40,41]. Potential protein–protein interactions of these genes, like COL3A1–FBN1 or COL5A2–FBN, have been described [32].

Collagen-encoding genes are the most studied in SCAD pathogenesis, especially in familiar cases. Many genetic variants have been found in these genes, ranging from rare early lethal phenotypes to common subclinical aspects. Collagens are a family of extracellular matrix proteins essential for normal tissue architecture, cell migration, and cell adhesion. A collagen molecule is formed by three alpha polypeptide chains that make up a triple helix [31,32]. Tarr et al. [31] identified likely pathogenetic variants in exon 42 and exon 51 of COL4A1 gene (which encodes for collagen type IV alpha 1 chain) in a young woman suffering for pregnancy-associated SCAD and in another young woman who experienced multiple SCAD events in her life. The same authors [31] identified another pathogenetic variant in COL4A4 gene (which encodes the collagen type IV alpha 4 chain) in a patient with Alport syndrome complicated by a SCAD event. In the same cohort, two other carriers of rare heterozygotic variants in COL4A4 were found: one likely pathogenetic variant and one splice-altering variant. They also showed that null COL3A1 variants may have an important role in SCAD, with possible treatment implications, thanks to a tailored prescription of β-blockers in SCAD patients with COL3A1 mutation [31]. COL3A1 gene (which encodes for collagen type III alpha 1 chain) variants have been found in more than 96% of patients with vascular Ehlers–Danlos syndrome, who often experience SCAD events due to arterial dissection without evidence of previous aneurysmatic degeneration [31,36]. Of note, pathogenetic or likely pathogenetic variants in COL3A1 gene are the most frequent in sporadic SCAD patients [31,36]. It is reasonable to always include the analysis of COL3A1 gene in the genetic testing of a proband, especially for repeated SCAD episodes or in suspected familial heritage. Turley et al. [32] found likely pathogenetic variants in COL4A2 gene (which encodes the collagen type IV alpha 2 chain) in five families, with at least two distinct heterozygous missense variants.

Rare variants of Talin-1 (TLN-1) gene have been shown to contribute specifically to SCAD pathogenesis in both familiar and in sporadic cases [31,32]. Rare variants have a major effect in familial SCAD, but common variants are found in sporadic cases [31,32]. TLN-1 is a cytoplasmatic protein with a key role in focal adhesion complexes linking the actin cytoskeleton to the extracellular matrix and inducing integrin activation. Turley et al. [37] identified a rare heterozygous missense variant due to nucleotide transition resulting in an aminoacidic substitution (p.A2013T) in a highly conserved β-integrin-binding domain of TLN-1 gene in familial SCAD. The same authors identified another nine additional rare heterozygous missense variants in TLN-1 gene in ten sporadic SCAD cases [37].

The SNP rs9349379 of the phosphatase and actin regulator 1 (PHACTR1) gene causes arterial wall pathologies, giving an increased risk not only od SCAD but also of spontaneous cervical artery dissection, migraine, and fibromuscular dysplasia [42,43]. This common variant downregulates the expression of endothelin-1 gene at its promotor so that local endothelin-1 levels are significantly lower. The SNP rs9349379 gives an almost 70% greater risk of SCAD compared to general population [42,43].

Specific FBN1 gene variants are known to cause Marfan syndrome [31,32,35]. Likely pathogenetic variants for SCAD in this gene were seen in two female cases, both lacking a previous diagnosis of Marfan syndrome [31,32].

Another likely pathogenic missense variant was identified in the aldehyde dehydrogenase 18 family member A1 (ALDH18A1) gene responsible for a rare cutis laxa syndrome [31]. Cutis laxa syndromes are rare heterogeneous genetic CTD disorders with common cardiovascular complications, especially when linked to autosomal recessive mutations. These patients usually present severe lax and wrinkled skin, skeletal anomalies, arterial tortuosity, arterial aneurysms, and variable intellectual disabilities [44].

Potentially, two likely pathogenic variants were identified in activin A receptor type 1 gene (ACVR1) in two SCAD cases, even though both patients had mutations in other genes, like ALDH18A1 gene. ACVR1 encodes for a receptor of bone morphogenetic proteins, which may have a role in vascular homeostasis [31]. These two variants need to be studied further to assess their association with SCAD.

Saw et al. [45] revealed an association for the variant rs12740679 in a disintegrin-and-metalloproteinase-with-thrombospondin-motifs-like (ADAMTSL4) gene with SCAD [45]. This gene encodes for an extracellular matrix protein, which binds FBN-1 and promotes the formation of microfibrils [35,38,45].

Recently, Bai et al. [46] described a rare case of pregnancy-associated SCAD in a 30-year-old woman in the early postpartum period. This patient had three different SCAD events in three different points of her coronaries within 10 days after the baby delivery. These authors detected, for the first time, a heterozygous missense variant, c.4574 C > T (p.Arg1438Cys), in the neurogenic locus notch homolog protein 1 (NOTCH1) gene. NOTCH1 is a large transmembrane protein with a specific signalling pathway conserved across species which plays a central role in vascular smooth muscle cell apoptosis [46].

Similarly, Vandeloo et al. [47] documented the first missense mutation, c.1082A > C (p.[Asn361Thr]), in SMAD2 gene in a case of sporadic SCAD. This variant was found in a 52-year-old man who experienced a SCAD episode after lifting heavy weights [47].

Lastly, Solomonica et al. [41] described in familial SCAD a novel pathogenetic mutation, c.860 G > A p.Arg287Gln, in exon 6 of SMAD3 gene. This mutation disrupts a well-preserved domain of the protein. Several members of the family were diagnosticated with Loeys–Dietz syndrome type 3 complicated by numerous and recurrent SCAD episodes [41].

To conclude, further analyses in larger cohorts are required in order to completely understand the genetic architecture of SCAD pathogenesis and to clearly define the overlap between SCAD, CTD, and vasculopathies.

## 4. Diagnostic Criteria and Current Therapeutic Management

Coronary angiography is the gold standard for SCAD diagnosis [8]. However, a simple two-dimensional exam is not always sufficient to satisfy diagnostic doubt, and adjunctive evaluation through optical coherence tomography (OCT) should be performed. Usually, SCAD involves a single coronary artery and a single artery’s segment, although multidistrict involvement has been described [14]. The main involved artery is the LAD, followed by the left circumflex and right coronary arteries [14]. Saw et al. [15] found a mean SCAD length of 33.2 mm. According to Yip-Saw classification, four main types of angiographic SCAD have been described [8,14,48,49]; (i) type 1, accounting for fewer than 1/3 of SCAD cases, is characterized by the distinct evidence of the false and true lumen after contrast medium passage. Type 1 SCAD has an evolving and progressive nature, although it is associated with a poor clinical progression and/or post-percutaneous coronary intervention (PCI) complications [48]; (ii) type 2 is the most frequent angiographic SCAD, and it is characterized by a variable narrowing of interested arteries, both in terms of length and diameter involvement. According to the latter, type 2a SCAD is characterized by a normal reperfused vessel distally to the false lumen; and type 2b is characterized by distal segments narrowing extension. Type 2 is the most frequent SCAD, but also the most angiographically missed [30]; (iii) type 3 is comparable to focal or tubular atherosclerotic lesions and not distinguishable from them (Table 1). The difference between the type 2 and 3 depends on the appearance of intramural hematoma [30]. Type 4 SCAD has been recently proposed and it is characterized by total vessel occlusion. It often involves small distal vessels, and a diagnosis of thromboembolic occlusion has to be carried out [50].

There are several angiographic findings associated with SCAD. In particular, SCAD is associated with coronary tortuosity and the site of the myocardial bridge. Differently from atherosclerotic lesions, SCAD mainly involves distal segments. Side branches often represent the starting and/or ending zone of the tear, particularly LAD side branches. In case of doubting coronary angiography, intracoronary imaging is required. OCT is preferred to IVUS due to its better spatial resolution [8,14,48,49]. OCT should be evaluated at the moment of the exam, and it allows for a precise visualization of true and false lumen, the beginning of the tear, and the severity of true lumen compression, and it is important to guiding the stent positioning during PCI, avoiding the false lumen stenting [8,51].

The role of intravascular imaging is increasing, particularly in doubtful cases. In this regard, IVUS and OCT may represent useful tools by which to make diagnosis. These techniques may be preferred to study SCAD of the proximal section of the main vessels. They can also be used to guide PCI. The use of OCT and IVUS may be related to dissection extension, iatrogenic new dissections, and vessel occlusion [52,53,54].

OCT provides high resolution images. It is characterized by an optical fiber positioned inside a catheter and using infrared light. This technique needs the use of contrast medium, which is responsible, together with the infrared light, for obtaining high-resolution images characterized by different color tones according to the tissue composition. It offers a high tissue characterization of coronary artery wall layers. This is important in identifying the intima layer and the pathognomonic sign of SCAD, that is, the intramural hematoma, with false lumen [52,53,54].

IVUS is an intravascular imaging method based on the use of an ultrasound transducer positioned at the end of the catheter. It does need contrast medium, and it allows for the visualization of intramural hematoma and the presence of false lumen. The spatial resolution is lower compared to OCT, and the grayscale may be insufficient to differentiate hematoma from a lipid-rich plaque [52,53,54].

Numasawa et al. [55] used the IVUS technique to diagnose SCAD and guide the positioning of the guide wire in the true lumen, avoiding the false lumen.

Also, Kano et al. [56] used the IVUS technique to detect SCAD in the proximal left descending artery and to insert the guide wire in the true lumen, allowing for stent positioning.

In order to decide the approach of treatment, distinguishing high-risk patients from lower-risk patients is pivotal to choosing the right treatment. Conservative treatment with medical therapy is the cornerstone of SCAD treatment. This is due to the observation that invasive treatment with PCI may be associated with an iatrogenic dissection or with a further extension of SCAD and repercussion on the ischemia severity. Moreover, PCI does not protect against recurrent SCAD. Mahmoud et al. [57] reported worse outcomes in patients with NSTEMI treated with PCI, confirming that lower in-hospital mortality was associated with a reduced use of PCI. Bocchino et al. [58] demonstrated that major adverse cardiovascular events (MACE) occurred more during the in-hospital period than in the following periods, without differences between conservative or invasive management. However, the conservative management was associated with a lower target vessel revascularization rate [59]. For this reason, the correct stratification and selection of patients is fundamental. In this regard, Yong et al. [60] demonstrated that the assessment of ischemia severity through non-invasive techniques represented a crucial mechanism by which to select treatment strategy. In fact, a reduction of myocardial infarction and MACE, but not death, has been reported in patients with severe and moderate ischemia managed with revascularization.

Patients are defined at high-risk in the case of recurrent or incessant chest pain, despite antianginal therapy; cardiogenic shock; the presence of ventricular tachycardia or fibrillation; and left main arteries involvement [14,58]. In high-risk patients, PCI is indicated in the case of isolated left main dissection and in the case of a recurrent or incessant chest pain in the absence of hemodynamic instability. Isogai et al. [61] identified several features that are associated with an invasive treatment compared to a conservative therapy. In particular, inferior and anterior STEMI and cardiogenic shock have been associated with revascularization [61]. However, in the case of more extended involvement of LAD and/or the circumflex artery, ostial LAD, the presence of at least 2 SCADs, and young age, a coronary artery bypass graft (CABG) is preferred, and it is associated with optimal outcome [58]. Lobo et al. [17] reported that PCI treatment of STEMI patients with SCAD was associated with positive 3-years survival. In the studied population, left main arteries involvement was frequent among STEMI patients with SCAD. Moreover, thrombolysis in myocardial infarction (TIMI) flow after procedure was often lower for patients with SCAD-related STEMI than atherosclerosis-related STEMI. Moreover, in SCAD patients with STEMI, the use of a mechanical circulatory support should be considered due to the high incidence of a cardiogenic shock. Kotecha et al. [62] confirmed that high-risk patients may benefit from invasive strategy, which is characterized by a higher complication rate and a more-numerous stents use with a good medium-term outcome.

PCI is indicated only in cases of SCAD with symptoms of a myocardial ischemia, i.e., a large area of myocardium in jeopardy and reduced antegrade flow. CABG is indicated only when SCAD affects the left main vessel or two proximal vessels with symptoms of myocardial ischemia, but PCI is not feasible or unsuccessful [1]. PCI demonstrated a high-risk of complications involving up to 40% of patients [1,63]. On the other hand, CABG has favorable early clinical outcomes despite a significant rate of graft occlusion at 5 years. This has been explained both because CABG on a dissected artery promotes anastomosis failure and because spontaneous healing of the dissection may restore the flow in the original vessel. For these reasons, vein grafts should be preferred to preserve the future use of arterial conduits [1]. The American Heart Association/American College of Cardiology’s (AHA/ACC) scientific statement for SCAD proposed additional invasive treatment options, such as the aspiration technique and the cutting balloon (CB) angioplasty [16]. The latter allows for communication between true and false lumen, although it has a risk of coronary rupture. The aspiration and the CB techniques have been reported in the literature as possible and valid alternative treatment modalities for SCAD [64,65].

Conservative therapy is the cornerstone of SCAD treatment. However, an important topic that deserves further study is the duration of antiplatelet therapy, especially when the patient with SCAD has not performed PCI. The major post-SCAD follow-up registers show a mostly benign prognosis of the disease, with a survival of 10 years >90% [66,67,68,69]. However, SCAD tends to recur, and this is the main long-term complication that must guide the decision of the therapy duration. The recurrence is higher during the first month after SCAD and, for this reason, a dual antiplatelet therapy (DAPT) strategy, preferably acetylsalicylic acid and clopidogrel, is encouraged for the first month [48]. If no high-risk elements are presented, the use of acetylsalicylic acid alone would be more cautious [68]. The use of dual antiplatelet therapy is a main issue in patients who did not undergo revascularization. Currently, there is not a standardized protocol regarding the need for DAPT and its duration. For this reason, the approach is subject to variance according to the protocol of the specific medical center considered [14]. Cerrato et al. [70] reported that SCAD patients conservatively treated with DAPT showed a higher rate of MACE, in particular unplanned PCI, and non-fatal myocardial infarction compared to patients treated with a single antiplatelet therapy.

In summary, the topic of medical versus invasive treatment of SCAD has been recognized as a “gap in evidence” by the latest guidelines and consensus paper [2,8]. In general, the target of SCAD treatment is to preserve coronary flow and stop the extension of hematoma [68]. According to recent studies, stent placement and fibrinolytic therapy may contribute to the expansion of hematoma, while the use of a DAPT would avoid the formation of a thrombus inside the false lumen [2,68,71]. In fact, some authors argue that SCAD itself may hyperactivate the immune system through yjr inflammation mechanism, generating a state of hypercoagulability [68,72]. Studies with OCT have demonstrated an effective low prevalence of thrombosis [68,73].

Although late MACE, such as SCAD recurrence and myocardial infarction, may occur, medical treatment is associated with a positive clinical outcome and dissection healing [66,69]. Beta-blockers are associated with reduced risk of SCAD recurrence [59]. Currently, the randomized clinical trial BA-SCAD (beta-blockers and antiplatelet agents in patients with spontaneous coronary artery dissection) [74] is evaluating the efficacy of beta-blockers, in association with 1-month or 12-month antiplatelet therapy, in terms of myocardial infarction, death from coronary revascularization, stroke, unplanned hospitalization for ACS or heart failure, and recurrent SCAD at 1 year. Lindhal et al. [75] found that the inhibition of renin angiotensin aldosterone system (RAAS) is associated with MACE reduction at 1 year. Statins should be designated to patients with dyslipidemia because their use has been associated with SCAD recurrence [67]. Saw et al. [68,76] reported a significative reduction in SCAD recurrence with beta-blockers therapy because of their shear stress reduction power. However, they must be used with caution, especially if SCAD involves the right coronary, in order to avoid excessive bradycardia and hypotension [68].

A summary of the treatment strategies, with indication and evidence, has been reported in Table 2.

Recently, in a meta-regression analysis, Mele et al. [77] demonstrated that the use of DAPT had a borderline association with SCAD recurrence, while the use of acetylsalicylic acid was associated with lower angina hospital readmission. The other drugs, such as statins, beta-blockers, and RAAS inhibitor, may be not associated with long-term outcome in patients with SCAD.

## 5. Conclusions

In recent years, the concept of ischemic heart disease has evolved. Interest in the macroscopic mechanism of atherosclerosis has shifted towards more microscopic mechanisms [78,79,80], along with an increase in the diagnosis of MINOCA [81,82,83]. SCAD is an interesting pathophysiological example of MINOCA, which remains often underdiagnosed. Despite a good overall survival rate, this condition seems common and potentially malignant because of recurrences and the possibility that it might cause several complications, such as sudden cardiac death and cardiogenic shock [84]. Randomized trials are awaited to fill the current gaps in SCAD treatment approach. Currently, the guidelines [1] recommend a non-invasive treatment based on the use of antiplatelets agents and clinical observation. However, sometimes, an interventional approach is preferred or mandatory, as in case of complications and myocardial ischemia persistence.

## 6. Future Directions

The current gap regarding the management of SCAD is mainly represented by the necessity to understand how to make the correct diagnosis and how to improve the treatment in order to prevent recurrencies. Moreover, given the particular pathophysiology of SCAD, a correct approach to this condition cannot ignore the genetic background [85,86]. Many patients are particularly exposed to SCAD due to genetic reasons, sometimes identifiable and sometimes unknown. The improvement of knowledge regarding genetic susceptibility to SCAD may improve its management, allowing for the identification of patients who are more exposed to this condition and patients with a high risk of recurrencies [85,86].

## Figures and Tables

**Table 1:** Angiographic classification of spontaneous coronary artery dissection according to Yip and Saw classification.

SCAD Type	Frequency	Features
**Type 1**	Around 29% of cases	It is characterized by the distinct evidence of the false and true lumen after contrast medium passage. It can be evidenced as contrast medium staining and/or multiple radiolucent lumens. It has an evolving and progressive nature, although it is associated with a poor clinical progression and/or post PCI complications.
**Type 2**	Around 67% of cases	It is characterized by variable narrowing of interested arteries, both in terms of length and diameters. Type 2a SCAD is characterized by a normal reperfused vessel distally to the false lumen; while type 2b is characterized by distal segments narrowing extension
**Type 3**	Around 4% of cases	It is comparable to focal or tubular atherosclerotic lesions and not distinguishable from them.
**Type 4**		It is characterized by total vessel occlusion. It often involves small distal vessels, and a diagnosis of thromboembolic occlusion has to be carried out.

SCAD: spontaneous coronary artery dissection; PCI: percutaneous coronary intervention.

**Table 2:** Treatment approaches to spontaneous coronary artery dissection. In the following table, the different treatment approaches (conservative medical therapy, PCI, and CABG), with ideal candidate patients and evidence, have been reported.

Type of Treatment	Candidate Patients	Evidence
**Medical Therapy**	(1) Cornerstone treatment in low-risk patients	MACE occurred more during the in-hospital period, without differences between conservative and invasive management [58]. Conservative management is associated with a lower target vessel revascularization rate [59]. DAPT strategy, preferably acetylsalicylic acid and clopidogrel, is encouraged for the first month because recurrence is higher [48]. If no high-risk elements are present, the use of acetylsalicylic acid alone would be more cautionary [68]. Statins should be destinated to patients with dyslipidemia because their use has been associated with SCAD recurrence [67]. Reduction in SCAD recurrence with beta-blockers therapy because of their shear-stress reduction power [68,76]. RAAS inhibition is associated with MACE reduction at 1 year [75].
**PCI**	(1) Patients with symptoms of ischemia, a large area of myocardium in jeopardy, and reduced antegrade flow (2) High-risk patients in case of isolated left main dissection (i.e., hemodynamic instability) (3) Patients with recurrent or incessant chest pain	Reduction of myocardial infarction and MACE in patients with severe and moderate ischemia managed with revascularization [60]. Better outcome in cases of inferior and anterior STEMI or cardiogenic shock managed with revascularization [61]. PCI treatment of STEMI patients with SCAD, especially with left main involvement, was associated with positive 3-years survival [17]. High-risk patients may benefit from an invasive strategy with a good medium-term outcome, despite higher complication rate [62]. PCI demonstrated a high-risk of complications involving up to 40% of patients, and it does not protect by recurrencies [1,63].
**CABG**	(1) Patients with the left main vessel or two proximal vessels with symptoms of ischemia and PCI not feasible/unsuccessful (2) Extensive involvement of LAD and/or circumflex artery; ostial LAD; presence of at least 2 SCADs and young age	CABG has favorable early clinical outcomes, despite a significant rate of graft occlusion at 5 years (anastomosis failure and spontaneous healing of the dissection) [1].

PCI: percutaneous coronary intervention; CABG: coronary artery bypass graft; DAPT: dual antiplatelet therapy; RAAS: renin–angiotensin–aldosterone system; MACE: major adverse cardiovascular events; STEMI: ST elevation myocardial infarction; LAD: left anterior descending artery.

## Data Availability

Not applicable.
